# Bilateral Multifocal Choroidal Osteoma with Choroidal Neovascularization

**DOI:** 10.1155/2015/346415

**Published:** 2015-12-06

**Authors:** Mousavi MirNaghi, Shoeibi Nasser, Hosseini SeyedehMaryam, Saadat Targhi Ali

**Affiliations:** ^1^Retina Research Center, Mashhad University of Medical Sciences, Mashhad, Iran; ^2^Eye Research Center, Mashhad University of Medical Sciences, Mashhad, Iran

## Abstract

Choroidal osteoma has been reported to be unilateral in approximately 80% of cases diagnosed with this condition. Herein we report the clinical characteristics and multimodal imaging findings in a rare case of bilateral multifocal choroidal osteoma. A 32-year-old female presented with decreased visual acuity (VA) in the right eye (20/100), though she had normal VA (20/20) in the left eye. Ophthalmoscopy and multimodal imaging investigation revealed bilateral multifocal choroidal osteoma complicated by choroidal neovascularization (CNV) in the right eye. Following three injections of intravitreal bevacizumab (IVB) for CNV in the right eye, VA improved to 20/40.

## 1. Introduction

Choroidal osteoma is a rare benign ossifying choroidal tumor. While unilateral in 80% of cases, it is often seen in healthy young females in their twenties or thirties [1]. With almost half of the patients presenting with drastic decreased visual acuity (20/200 or worse), the condition may lead to complete visual loss mainly due to tumor growth, decalcification, and choroidal neovascularization (CNV) [2]. The tumor is often detected in the peripapillary zone, with a yellow-orange appearance [1–3].

We report here an interesting case in that it is bilateral with multiple tumoral foci. Also, the best corrected visual acuity (BCVA) had remained in the left eye whereas the right eye lost vision due to decalcification as well as CNV.

## 2. Case Report

**Figure 1:**
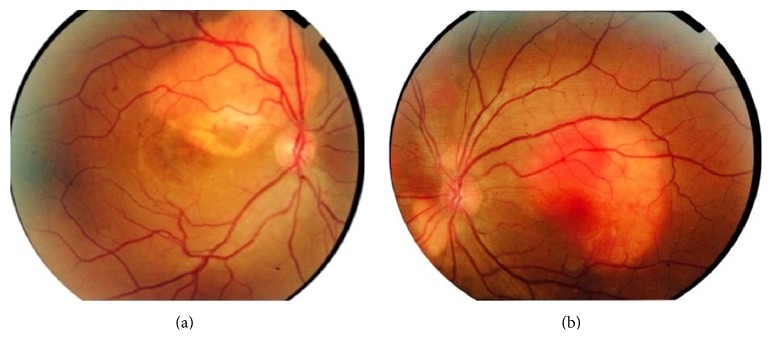
Fundus photography disclosed three foci of subretinal tumors in the left eye and large well-defined tumor in the macular area of the right eye with variable color due to decalcification, CNV, and SRF.

A 32-year-old female presented with decreased central vision in the right eye to our tertiary eye center (Khatam Eye Hospital) affiliated to Mashhad University of Medical Sciences. Institutional review board approval was obtained for this case report. Complete ophthalmic examination was done. BCVA was 20/100 on the right and 20/20 on the left one. Ophthalmoscopy revealed bilateral multifocal yellow-orange elevated choroidal masses with distinct borders at the posterior pole. The left eye was conspicuous in its macular tumor as well as juxtapapillary foci, indicative of multifocal nature of the lesion (Figure 1).

**Figure 2:**
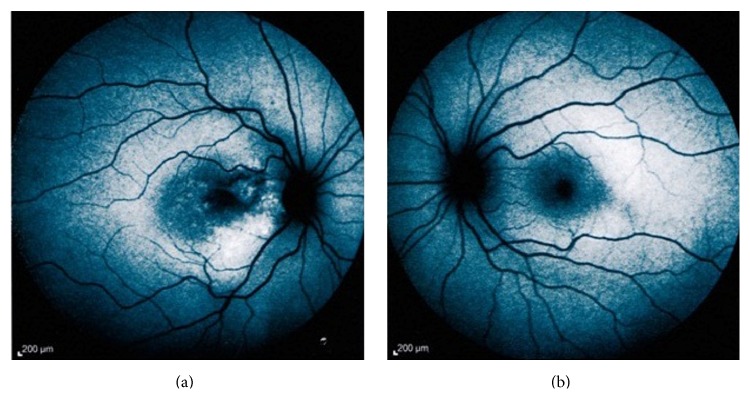
Fundus autofluorescence image shows variable hypo- and hyperautofluorescence in the right eye. In the left eye, isoautofluorescence of macula is evident.

**Figure 3:**
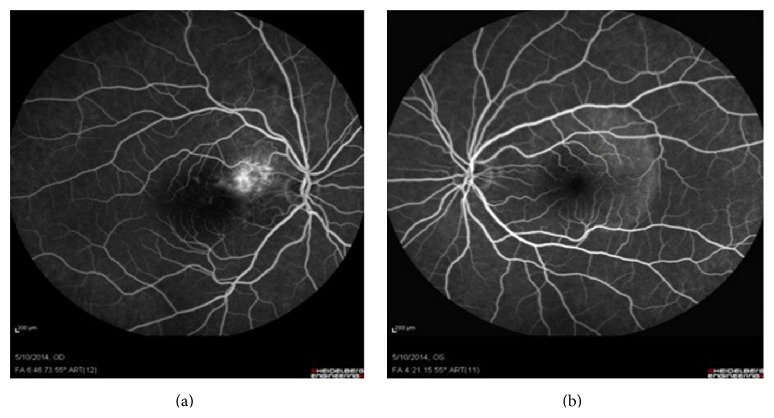
FAG; hyperfluorescent area in the right eye is CNV membrane, and hyperfluorescence over the mass in the left eye is window defect due to RPE and retinal atrophy.

**Figure 4:**
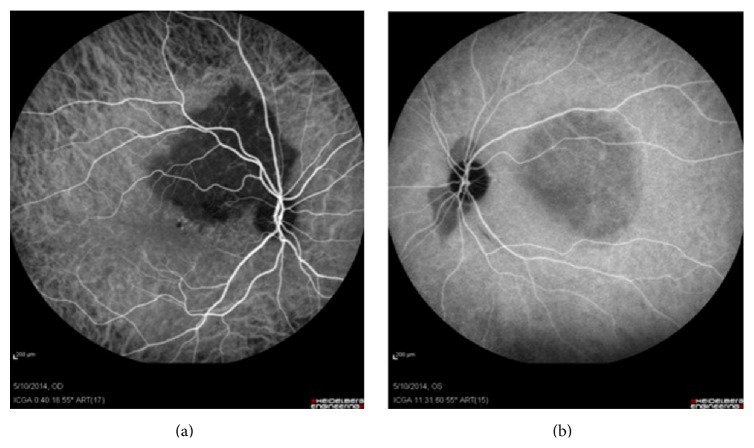
ICG shows well-defined hypofluorescent lesions compatible with tumor in early to mid-phase.

Fundus autofluorescence (FAF) images revealed the calcified parts of the tumor to be isoautofluorescent (AF) or mild hyper-AF on the left, whereas the lesion was variable (hypo-hyper AF) on the right side, possibly due to RPE changes, which in turn stemmed from decalcification, CNV, and subretinal fluid (SRF) (Figure 2). Fluorescein angiography (FAG) demonstrated variable hyperfluorescence of the tumor on both sides as well as CNV in the right eye (Figure 3). Indocyanine green angiography (ICG), in early to mid-phase, showed the well-defined tumor to be hypofluorescent with CNV on the right side (Figure 4).

**Figure 5:**
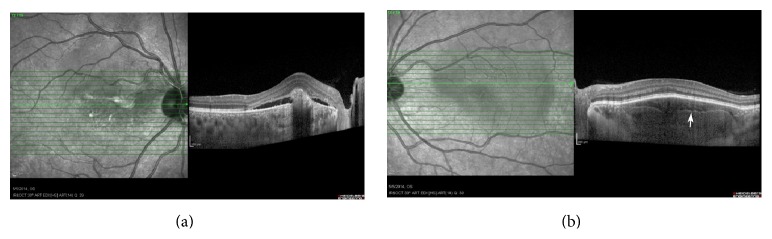
(a) EDI-OCT revealed outer retinal abnormality and SRF due to CNV overlying the tumor in the right eye. (b) Sponge-like choroidal lesion with horizontal hyperreflective lines (arrow) inside the tumor, thinning of choriocapillaries, and large choroidal vessels in the left eye (b).

**Figure 6:**
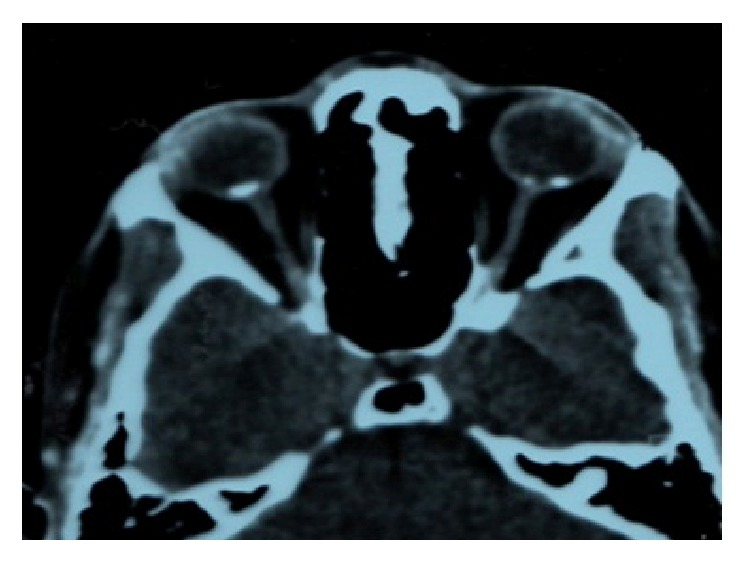
Orbital computerized tomography (CT) scans show hyperdense plaques, with bone-like homogeneity in the posterior pole. Two plaques are seen in the left eye.

B-scan ultrasonography (NIDEK Echo scan US-4000, Gamagori, Japan) indicated hyperechogenicity of the tumor on both sides. Enhanced depth imaging optical coherence tomography (EDI-OCT) (Spectralis HRA + OCT, Heidelberg Engineering, Heidelberg, Germany) disclosed the thinning of choriocapillaries as well as large choroidal vessels, which were invisible in the calcified parts. Outer retinal abnormalities were attributed to CNV in the right eye. These regions also showed the disturbance of the outer retinal layers whereas the inner retina turned out to be entirely intact. The tumor displayed smooth undulating surface with horizontal layered hyperreflective lines in the tumor (Figure 5). Orbital computerized tomography (CT) scan showed hyperdense plaques, with bone like homogeneity in the posterior pole. Two plaques were seen in the left eye (Figure 6).

The final diagnosis of bilateral multifocal choroidal osteoma in association with CNV in the right eye was made based on multimodal imaging findings. The decalcified portion of tumor on the right was yellowish-white in color.

The safe conclusion pertaining to the above details was suggestive of the trend whereby tumor calcification and CNV had led to visual loss on the right eye. Thus, the patient received injections of intravitreal bevacizumab (Avastin, Genentech Inc., San Francisco, CA) in the right eye, which subsequently improved the BCVA to 20/40. This BCVA sustained during nine months of follow-up.

## 3. Discussion

This case is of considerable interest owing to the bilaterality as well as multifocality of the choroidal osteoma. The former can be found in 20% of cases whereas the latter is quite a rare condition (3 foci). The diagnosis is made when an amelanotic subretinal mass is detected often at the peripapillary area and adjacent to the posterior pole. A variety of multimodal imaging procedures including orbital CT scan, ultrasonography, FAG, ICG, and OCT [1, 2] have been used as in our case.

Pellegrini et al. highlighted EDI-OCT features of osteoma in 7 eyes, reporting a typically sponge-like tumor with the multiple intralesional layers, choriocapillaries thinning, different abnormalities in the external limiting membrane, photoreceptor layers, and retinal pigment epithelium (RPE) [4].

The calcified parts of tumor reported to be isoautofluorescent in FAF [5].

The calcified region appeared yellow-orange as a result of RPE overlying the tumor remaining intact. The decalcified region was recognized as a yellow-white area, following the thinning as well as depigmentation of the overlying RPE. Osteoma often undergoes decalcification when growing (51% of cases with long-term follow-ups) [3]. Research investigating the long-term sequel reported visual acuities of 20/200 in 58% (36 eyes studied by Aylward et al.) and 56% (74 eyes investigated by Shields et al.) in a 10-year follow-up series [3, 6]. We only managed to follow the case in only 9 months, yet the time span seems too short to arrive at any definite conclusion. Vision loss occurs chiefly due to tumor growth, CNV, and decalcification resulting in changes in the photoreceptor layer or the RPE [2]. CNV membrane overlying choroidal osteoma has been found in 31% of cases by 5 years, 31–47% by 10 years, and 46–56% by 20 years [3].

In this case, the main cause of vision loss in the right eye was CNV membrane and tumor decalcification that resulted in outer retinal destruction. Decalcification did not occur in the left eye, so VA remained intact because of intact RPE and outer retina despite having a sizable tumor. Choroidal osteoma has eluded standard treatment modalities, though interventions are often aimed at pertinent complications, namely, CNV and subretinal fluid.

Certain modes of therapy have been used for CNV secondary to osteoma, ranging from photodynamic therapy (PDT) [7] to transpupillary thermotherapy (TTT) [8] and surgical excision [9]. Yet they all seem to fail rigorous tests establishing their efficacy. Mansour et al. reported the efficacy of antivascular endothelial growth factor therapy for CNV secondary to choroidal osteoma with modest visual gain [10]. Regarding the report by Mansour et al. [10] and unavailability of PDT in our center, we decided to treat this case, administering three consecutive monthly intravitreal injections of bevacizumab (Avastin, Genentech Inc., San Francisco, CA). As a result, BCVA improved from 20/100 to 20/40 after injection, having sustained during the nine months of follow-up. Residual SRF was detected in our last F/U; yet against our instructions, the patient refused to receive additional injections.

In conclusion, although choroidal osteoma is a benign tumor, it can lead to profound visual disturbance due to CNV, decalcification, and RPE abnormality. We should be aware of possible bilaterality and multifocality in these cases. It seems that bevacizumab is an effective treatment for the CNV secondary to choroidal osteoma. Long-term follow-up is necessary for early detection and treatment of possible CNV.
